# Evidence of hemolysis in pigs infected with highly virulent African swine fever virus

**DOI:** 10.14202/vetworld.2016.1413-1419

**Published:** 2016-12-14

**Authors:** Zaven Karalyan, Hovakim Zakaryan, Elina Arakelova, Violeta Aivazyan, Marina Tatoyan, Armen Kotsinyan, Roza Izmailyan, Elena Karalova

**Affiliations:** 1 Laboratory of Cell Biology and Virology, Institute of Molecular Biology of NAS RA, 7 Hasratyan Street, 0014 Yerevan, Armenia; 2 Laboratory of Human Genomics and Immunomics, Institute of Molecular Biology of NAS RA, 7 Hasratyan Street, 0014 Yerevan, Armenia

**Keywords:** African swine fever virus, bilirubinemia, bilirubinuria, erythropoietin, hemolysis, proteinuria

## Abstract

**Aim:**

The research was conducted to understand more profoundly the pathogenetic aspects of the acute form of the African swine fever (ASF).

**Materials and Methods:**

A total of 10 pigs were inoculated with ASF virus (ASFV) (genotype II) in the study of the red blood cells (RBCs), blood and urine biochemistry in the dynamics of disease.

**Results:**

The major hematological differences observed in ASFV infected pigs were that the mean corpuscular volume, mean corpuscular hemoglobin, and hematocrits were significantly decreased compared to controls, and the levels of erythropoietin were significantly increased. Also were detected the trends of decrease in RBC count at terminal stages of ASF. Analysis of blood biochemistry revealed that during ASF development, besides bilirubinemia significantly elevated levels of lactate dehydrogenase, and aspartate aminotransferase were detected. Analysis of urine biochemistry revealed the presence of bilirubinuria, proteinuria during ASF development. Proteinuria, especially at late stages of the disease reflects a severe kidney damage possible glomerulonefritis.

**Conclusion:**

The results of this study indicate the characteristics of developing hemolytic anemia observed in acute ASF (genotype II).

## Introduction

Since its introduction into Georgia, African swine fever virus (ASFV) has rapidly spread into Armenia, vast areas of western and southern Russia, and also Ukraine and Belarus [1]. ASF is a highly lethal disease of domestic pigs caused by the only known DNA arbovirus. In pigs, ASFV replicates in cells of the mononuclear phagocyte system, predominantly monocytes, and macrophages. Several other cell types can be infected, especially in the later stages of the disease. Many scientists consider that the massive destruction of macrophages plays a major role in pathogenesis, particularly in the impaired hemostasis due to the release of active substances such as cytokines and complement factors however despite intensive research efforts, most of these pathogenetical aspects are still far from being understood [2].

Serum biochemistry standards in veterinary are now available from many commercial sources. They are widely used by biochemists and clinicians because of their great convenience and informativeness. Hematological and biochemical profiles are commonly performed in veterinarian medicine for a variety of reasons: As screening tests on clinically healthy animals, to diagnose a disease and to assess its severity and consequences, to formulate a prognosis and to monitor the response of therapy or the progression of the disease. Despite their clinical importance, interpretation is sometimes difficult as many factors can significantly modify the experimental parameters, such as gender, age, breed, diet, environmental conditions, samples management, laboratorial procedures, health status, and subclinical diseases [3].

In the view of the mentioned points, the purpose of this study is to determine what pathological changes occur in red blood cell (RBC), blood, serum, and urine clinical biochemistry as well as urine sediment analyses in pigs with acute ASF. Another objective is the interpretation of the laboratorial data in general pathology of acute ASF.

## Materials and Methods

### Ethical approval

All procedures of sampling collection were performed strictly as specified by Independent Ethics, Committee of the Institute of Molecular Biology of NAS, IRB00004079 with minimal stress to animals.

### Virus

Infections were carried out using ASFV (genotype II) distributed in Republic of Armenia and Republic of Georgia [4]. The titer of ASFV for each intramuscular injection was 10^4^ hemadsorbing doses (HAD50)/ml. Virus titration was done as described previously and expressed as Log 10 HAD50/ml for non-adapted cells [5].

### Animals

In our study, 10 healthy pigs (Landrace breed) of the same age (3-month-old) and weight (30-33 kg) were used for infection and two for control.

Preinoculated blood samples were taken from all pigs to obtain control values. Considerable care was taken in the collection of the blood samples to avoid hemolysis and tissue contamination. The blood samples were obtained from the anterior vena cava.

About 10 pigs were infected by intramuscular injection, and two pigs (intramuscular injection of physiological solution – 1.0 ml) were used as an uninfected control. Animal care and euthanasia were done according to the AVMA Guidelines on Euthanasia, and Local Guideline for Animal Care and use (Institutional Review Board/Independent Ethics Committee of the Institute of Molecular Biology of NAS, IRB00004079). Carbon dioxide inhalation (75-80% carbon dioxide for 20 min) was used to euthanatize infected and control animals after 7 days post-infection (DPI).

### Analyses of peripheral blood

RBC count, hematocrit (Hct), mean corpuscular volume (MCV), mean corpuscular hemoglobin (Hb) (MCH), and Hb concentration was prepared in ethylenediaminetetraacetic acid. Whole-blood samples were measured using an automatic blood cell counter, Sysmex XS 1000i. For fine Hb measurements, the specimens were analyzed on an automated microscope system employing television scanning according to the Soret technique [6,7].

### Blood collection and enzyme-linked immunosorbent assay (ELISA)

From 1 to 7 DPI, blood samples were collected from the swine ophthalmic venous sinus and for controls all samples were collected at day 0 prior to virus inoculation. The levels of erythropoietin (Epo) in plasma samples were measured by an ELISA kit (MyBioSource; Porcine Epo, MBS269716).

### Nucleated RBC (NRBC) count and units of reporting

Manual microscopy was used to count the NRBC for which a drop of blood was smeared over a glass slide and stained by the Giemsa modified solution according to manufacturer’s description, Sigma-Aldrich. The hematology laboratory results were reported as NRBC per 100 white blood cells and converted in an absolute number of cells in mm^3^ [8].

### Serum biochemistry

For clinical chemistry, whole blood samples without additives were allowed to clot at room temperature for several hours and centrifuged for 5 min at 1500 rpm. The levels of bilirubin in serum were measured using a biochemical analyzer (COBAS Integra 400 analyzer) with standard methods. Enzymatic abnormalities were evaluated by measurement of gamma-glutamyltransferase, lactate dehydrogenase (LDH) (U/L), alanine aminotransferase (ALT), and aspartate aminotransferase (AST).

### Urine chemistry

Urine chemistry was evaluated using Test Strips from Roche Diagnostics (on Urisys-1100 analyzer) and freshly expressed urine.

### Tissue samples

Samples from livers were fixed in 10% buffered formalin solution (pH 7.2) for 24 h. After fixation, the samples were dehydrated through a graded series of alcohols, washed with xylol and embedded in paraffin wax by a routine technique for light microscopy. For structural analysis, wax-embedded samples were cut (Microm HM 355, 5 Lm) and stained with hematoxylin and eosin in accordance with the manufacturer’s protocol (Sigma-Aldrich, Germany). The histological examination was implemented using a light microscope.

### Statistical analysis

Statistical analyses were performed using the Student’s *t*-test and Mann–Whitney U-test. SPSS version 17.0 software package (SPSS Inc., Chicago, Illinois, USA).

## Results

### Experimental infection

The clinical signs of experimental infection have some differences from those in cited researches with Malawi’ 83 ASFV isolate [9] and our previous study [10].

The first clinical signs were observed at 2-3 DPI when all infected pigs demonstrated loss of appetite and depression. From 2 to 4 DPI, infected animals displayed hyperthermia with body temperature more than 40°C. Simultaneously, decreased activity in behavior, difficulties in breathing and reddening of the skin were detected. Bloody diarrhea (present only in 20% cases) and lethargy were seen at 5-6 DPI, and therefore, all infected animals were sacrificed according to guidelines at 7 DPI. Although infected animals were asymptomatic until 3 DPI, viremia was observed in all pigs starting from 1^st^ DPI and virus titer reached up to 5.0-5.5 log10 HAD50/ml at 7 DPI. These data conforms the clinical studies of ASF described previously [11,12].

### Hematological findings in ASFV infected pigs

As shown in Table-1, the major phenotypic hematological differences were observed in ASFV infected pigs: Such as RBC, MCV, MCH, and Hct were significantly lower in ASFV infected animals compare to controls. Whereas Epo levels in plasma of ASFV infected pigs were significantly higher (2^nd^ DPI tendency, 3^rd^ DPI significant) than that of the control animals. The two highest Epo levels, 8.5 and 8.8 ng/ml, were recorded on the terminal stage of ASF on day 6 and 7 post-infection. Although some differences occurred at 2-3 DPI (increased Epo level), Epo levels raised significantly at 3-4 DPI, and some evaluations show only tendency at 6-7 DPI (decreased MCV).

**Table-1:** Hematological findings in ASFV-infected pigs.

Indices	Control	1 DPI	2 DPI	3 DPI	4 DPI	5 DPI	6 DPI
RBC 10^6^/mm^3^	7.1±0.9	7.5±1.0	7.5±1.0	6.9±0.8	6.3±0.9	5.5±0.9**	4.9±0.8*
Reticulocyte (%)	0.98±0.1	0.2±0.01	0.2±0.01	1.4±0.01	7.9±0.5*	5.7±1.1*	16.2±1.2*
MCV (μm^3^)	6.8±0.3	7.1±0.9	6.3±1.0	6.2±1.0	6.2±0.8	6.2±0.8	6.1±0.7
Hct (%)	48±3.0	53±4.3	47±2.9	43±4.0	39±2.8**	34±2.9*	30±2.5*
MCH (pg)	18.2±2.1	21.2±2.2	20.1±2.7	16.8±2.5	17.6±1.8	16.5±2.2	15.2±1.9**
Hb (g/dl)	13.9±1.8	14.2±2.7	13.7±2.1	10.3±2.0	8.8±1.7***	7.6±1.5*	7.4±1.9*
Epo (ng/ml)	0.3±0.1	0.8±0.2	1.4±0.5**	3.0±0.9*	6.2±1.5*	5.9±1.8*	8.5±1.9*

* Significant compared with control (p<0.05-0.001),

** Number of inversions 2 (p<0.1),

*** Tendency (p<0.1).

MCV=Mean corpuscular volume, MCH=Mean corpuscular hemoglobin, Hb=Hemoglobin, Epo=Erythropoietin, DPI=Days post-infection, ASFV=African swine fever virus

**Figure-1:**
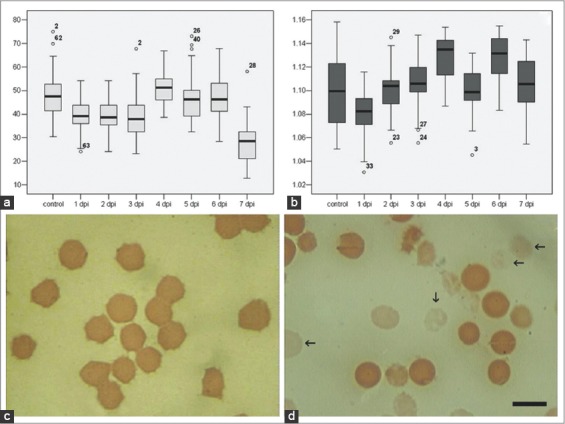
Erythrocytes at African swine fever: Red blood cell (RBC) size (a), excluded fragmentized cells, RBC density (b), excluded fragmentized cells and view in control (c) and pathology (d). Blood slides were stained by Giemsa modified solution. Cells were examined under the light microscope at 1250× magnification. Scale bar is 10 µm.

The observation of peripheral blood of uninfected pigs showed that the population of RBCs mainly consisted of mature erythrocytes and an insignificant number of reticulocytes. The average size of mature erythrocytes (normocytes) was 46.3±2.1 μm^2^, whereas the average diameter of a small number of erythrocytes considered as microcytes and macrocytes was respectively 20 (or less) and 63.5 μm^2^ (or greater). As acute ASFV infection progressed, the number of microcytes gradually increased, reaching 15-20% of the total RBCs at 7 DPI (Figure-1a). The density of an RBC of healthy pigs is about 1.1 g/cm^3^. At acute ASF there were no significant changes of this index (Figure-1b). Blood analysis (Figure-1d) at 6-7 DPI has been shown arising of “ghost” erythrocytes (were absent in healthy animals and early stage of ASF - Figure-1c).

**Figure-2:**
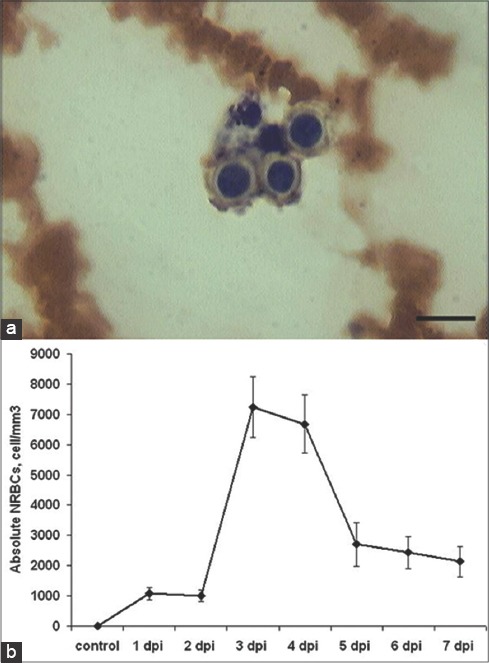
Nucleated red blood cells (NRBC) of African swine fever (ASF): NRBC at 4 days post-infection (a) blood slides were stained by Giemsa modified solution. Cells were examined under the light microscope at 1250× magnification. Scale bar is 10 µm. Absolute NRBCs in ASF dynamics (b), cell/mm^3^.

Interestingly at the early stages of infection, NRBCs appeared in the blood of infected animals (Figure-2a). The absolute NRBC count of pigs with ASF at 3-4 DPI was significantly higher compared to those in control and other days of disease (Figure-2b). The number of NRBCs did not exceed 0.5% of all RBCs.

### Biochemical profile and liver pathology

**Figure-3:**
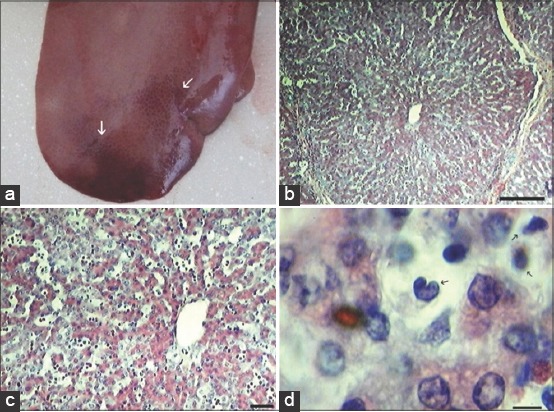
Liver histopathology: Subcapsular liver hemorrhages (white arrows) from 3 days post-infection (DPI) (a). Erythrocyte accumulation in the areas of liver parenchyma, at 6 DPI (b), scale bar is 250 µm, and (c) Scale bar is 100 µm. At late stage of African swine fever arise infiltrates of leukocytes in portal spaces and sinusoids at 6 DPI (d) arrows show massive leukocytic infiltrates (Scale bar is 10 µm).

At autopsy surface of the liver showed many subcapsular hemorrhages from 3 DPI (Figure-3a). Lesions in livers of pigs infected with ASFV consisted of degeneration and necrosis of parenchymal cells. Furthermore, there was erythrocyte accumulation in the areas of liver parenchyma (Figure-3b and c). At late stage of ASF infiltrates of leukocytes in portal spaces and sinusoids were arise (Figure-3d).

A biochemical profile, which included glucose, urea nitrogen, creatine kinase, creatinine, cholesterol, sodium, potassium, calcium, and phosphorus were done on all pigs either infected or control. Moreover, no significant differences (p<0.05) in these parameters were observed.

Serum bilirubin was determined in all 10 pigs with ASF and healthy controls. And shown that total bilirubin was increased significantly (p<0.01) till the end of disease (Table-2).

**Table-2:** Serum chemistry determinations for control and ASFV-infected pigs.

Parameters	Control	1 DPI	2 DPI	3 DPI	4 DPI	5 DPI	6 DPI
Bilirubin (µmol/L)	19.2±4.8	25.1±2.3	41±5.7*	51±7.3*	52±8.4*	62±9.1*	76±9.3*
Indirect (µmol/L)	16.1±3.2	22.8±3.0	27±4.9	36±5.6*	35±6.0*	43±7.2*	54±8.7*
Direct (µmol/L)	3.1±1.2	2.3±0.1	14±3.0*	15±3.9*	17±5.1*	19±4.0*	22±6.8*
AST (U/L)	44.5±7.2	122±26	230±51*	305±104*	826±299*	1302±552*	1194±392*
ALT (U/L)	32.2±8.9	38±9	40±12	32±10	55±13**	59±17**	48±9
LDH (U/L)	751±93.1	612±111	1018±322	1334±410*	3223±972*	2987±625*	1996±543*

* Significant compared with control (p<0.05-0.001),

** Number of inversions 2 (p<0.1). ASFV=African swine fever virus, DPI=Days post-infection, AST=Aspartate aminotransferase, ALT=Alanine aminotransferase, LDH=Lactate dehydrogenase

As follows from Table-2, during ASF development, besides bilirubinemia (including both fractions – direct and indirect) significantly elevated levels of AST, ALT and LDH also were detected.

### Urine chemistry and kidney pathology

Renal pathology at autopsy in pigs at the late stages of ASFV infection is well described in previous studies [13] by renal petechiae and/or diffused hemorrhages at the developed glomerulonephritis.

Changes observed in our study of the urine chemistry of pigs infected with ASFV are summarized in Table-3. Urine chemistry was done on ten pigs with ASF and was compared to controls (Table-3). Urine from pigs with ASF was generally clear and deep yellow up to amber in color. During ASF development, we observed the presence of blood in urine, bilirubinuria, proteinuria and changes in urine color (Table-3). Macroscopic hematuria was found in only one pig (10%) at the terminal stage of disease.

**Table-3:** Urine chemistry for pigs with ASF in disease dynamics.

Parameters	Control	1 DPI	2 DPI	3 DPI	4 DPI	5 DPI	6 DPI
Urobilinogen	Negative	Negative	Negative	Negative	Negative	Negative	Negative
Blood	Negative	+	+++*	+++*	+++*	+++*	+++*
Bilirubin	Negative	Negative	Negative	Negative	+*	+**	+*
Glucose	Negative	Negative	Negative	Negative	Negative	Negative	Negative
Protein (mg/L)	<150	152±19	420±45*	354±31*	435±54*	987±109*	5554±603*
Color	Pale yellow	Yellow	Yellow	Yellow	Dark yellow	Amber	Amber

* Significant compared with control (p<0.05-0.001),

** Number of inversions 2 (p<0.1). ASF=African swine fever, DPI=Days post-infection

## Discussion

The presence of NRBCs in the peripheral blood of adult pigs is strong evidence of marked disturbance in the blood production. Usually, the escape of nucleated blood cells into the human peripheral blood is indicative of a very strong stimulus that allows the release of these cells before they pass through the reticulocyte stage to become mature RBC. Okpara [14] suggest that severe anemia is a one of major stimulus in the release of immature cells into the peripheral blood. Furthermore, erythroblastemia has been reported in a wide variety of infections. It is usually considered to be part of leukemoid reaction in response to the infection [15]. The presence of erythrocyte “ghosts” in peripheral blood films indicates that the RBC lysed before blood film preparation [16].

Hyperbilirubinemia was a consistent finding in pigs with ASF. The occurrence of hyperbilirubinemia and increase in magnitude indicates a progressive increase in the serum bilirubin levels from early to late stages of the disease. Examination of the proportions of direct and indirect bilirubin indicates usually significant increases in both fractions (the averaged data presented in Table-1). It is well known that in certain syndromes (such as hemolysis) increased bilirubin formation rate in serum produce unconjugated hyperbilirubinemia. Elevated conjugated bilirubin levels usually indicate hepatobiliary pathology. It is also well known that intravascular hemolysis will increase the plasma bilirubin above the normal, but the serum bilirubin alone is therefore not a reliable index of the degree of hemolysis [17].

Epo mediates its effects on erythroid proliferation and differentiation through a number of different signaling pathways. Usually, it is produced in response to tissue hypoxia either due to increased tissue oxygen requirement or to decreased tissue oxygen delivery. One of the major determinants of tissue oxygen delivery is the Hb concentration in the blood. Consequently, in anemia caused by a primary dysfunction of red cell production or destruction, serum Epo titers usually increased above normal, generally in proportion to the severity of the anemia [18,19].

From medical studies we know, LDH is often used as a marker of tissue breakdown as LDH is abundant in RBCs and can function as a marker for hemolysis. Moreover, an elevation of the activity of LDH in serum can be an indicator for the severity of hemolysis [20,21]. Elevated levels of LDH are also indication of liver damage, which occurred at ASF.

Serum levels are mildly increased in extravascular hemolysis, such as immune hemolytic anemia, but are substantially elevated with intravascular hemolysis. Our data indicate that simple steady-state serum LDH is a convenient biomarker for the pathologic accumulation of Hb and its derivates in blood plasma. Hence, LDH, a readily available clinical laboratory test, appears to predict a direct hemolysis or syndrome of hemolysis associated endothelial dysfunction during ASF.

The elevation in both ALT (tendency) and AST was detected in the pigs with acute ASF. Both enzymes are present in the liver and are increased in serum during damage to hepatocytes. Increased ALT and AST is relatively specific for liver damage but also may originate from other tissues - notably cardiac and skeletal muscles [22].

The results of the urinalysis (Table-3) from pigs with ASF are consistent with an interpretation of hemolytic disease with the secondary liver disease. Since indirect or unconjugated bilirubin does not normally pass the glomerular filter, the bilirubinuria found in ASFV infected pigs is interpreted as conjugated. This correlates closely with high levels of direct or conjugated bilirubin in the serum of pigs with ASF. Conjugated bilirubinemia and bilirubinuria will accompany hemolytic disease if secondary liver disease is present [22]. The elevated bilirubin, both fractions conjugated and unconjugated, together with elevation of hepatic enzymes AST, ALT, and LDH, are also markers of liver damage and primarily suggest the existence of hepatitis. Similarly, the presence of bilirubin in urine is characteristic of hepatitis.

Urinalysis revealed mild proteinuria at 2 DPI and severe proteinuria at terminal stage of disease. Persistent and increased protein levels in the urine are abnormal. Renal loss of plasma proteins can contribute to several pathologies including hypoalbuminemia; alterations in coagulation factors, cellular immunity, hormonal status, etc. However, all are a sign of renal damage.

Simplistically anemia is a condition in which blood has a lower number of RBCs or contains low Hb concentrations in RBCs. Usually, anemia is caused by three main reasons: Blood loss, lack of RBC production, or high rates of RBC destruction. According to our results, intravascular RBC destruction and possible blood loss (at late stage of infection) could be reason for anemia in this study.

It is well known that lesions in animals with ASF include splenomegaly. The spleen is the majors its site for removal of damaged or hemolyzed erythrocytes [23]; hence, the splenomegaly observed in ASF could be presumed to be not only primary but also secondary to this process.

On the basis of obtained data, we can conclude that the pathology of red blood in ASF has a complex structure, and consists of two major components. The first component of the red blood pathology is erythroblastosis occurring from the first days of the disease. Its early appearance is a characteristic pathology of the central macrophage of erythroblastic island. A similar phenomenon is well described in literature [24], and we can reasonably suppose that the main reason of erythroblastosis is the direct lesion of these macrophages by the virus. The next etiological component in the pathology of red blood identified in our laboratory is intravascular hemolysis. It appears in the viremia caused by different viruses: Hepatitis C virus [25], cytomegalovirus [26], by some hemorrhagic fever viruses [27,28]. Causes of hemolysis in viral diseases may be the following factors: Pathological immune response, as a type of acute autoimmune reaction [29], direct or indirect action of the virus on erythrocytes [30], the incidence of precursors of erythropoiesis [31], etc. There are two-ways in which the direct effect of the virus on the RBCs can happen: Direct infection of erythrocytes and/or hemadsorption.

Another cause of hemolysis in ASF could be the hemadsorption of erythrocytes to infected cells as described in Breese and Hess [32]. Our studies of the direct effect of the virus on erythroid cells *in vitro* have shown that despite viral replication in culture, erythroid cells of primary culture bone marrow were refractory to its action compared to leukocytes and monocytes [33]. Therefore, we can assume that the pathology of the RBCs occurs only *in vivo*, in other words, it is the response to viral infection.

To explain the pathology, we must identify a specific pathology type in ASF - hypercytokinemia [34]. Hypercytokinemia occurs in response to sepsis (including viral pneumonias) resulting in an abnormally increase of primary proinflammatory cytokines of blood serum, tumor necrosis factor alpha and interleukin-1 (IL-1). It is known that the tumor necrosis factor and IL-1 β are inhibitors of Epo [35,36]. It is likely that the impact of proinflammatory cytokines led to the later increase of the level of Epo in the ASF.

Although this study is exploratory in nature, and certainly requires further validation in other ASFV-infected pigs group, the findings may be generalizable to the developing hemolytic processes. A common pathogenic feature of ASFV can described as an ability to disable the host immune response by attacking and manipulating cells that initiate the antiviral response. This pathology is characterized by marked replication of the virus as well as disregulation of the vascular system and lymphoid tissue [37]. In our study, we have demonstrated the characteristics of hemolytic anemia observed in acute ASF (genotype II). This study will not only contribute to further understanding of the mechanism of ASF pathogenesis and taking effective therapies, but also provide useful information and suggestions for other related research fields.

## Authors’ Contributions

ZK and EK designed the experiment. EA, VA, MT, AK and RI conducted the experiment. HZ did technical writing and revision of the manuscript. ZK and EK prepared the manuscript. All authors have read and approved the final version of the manuscript.

## References

[1] Oura C (2013). African swine fever virus:On the move and dangerous. Vet. Rec.

[2] Blome S, Gabriel C, Beer M (2013). Pathogenesis of African swine fever in domestic pigs and European wild boar. Virus Res.

[3] Muñoz A, Riber C, Trigo P, Castejón F (2012). Age-and gender-related variations in hematology, clinical biochemistry, and hormones in Spanish fillies and colts. Res. Vet. Sci.

[4] Rowlands R.J, Michaud V, Heath L, Hutchings G, Oura C, Vosloo W, Dwarka R, Onashvili T, Albina E, Dixon L.K (2008). African swine fever virus isolate, Georgia. Emerg. Infect. Dis.

[5] Enjuanes L, Carrascosa A.L, Moreno M.A, Viñuela E (1976). Titration of African swine fever (ASF) virus. J. Gen. Virol.

[6] Bacus J.W (1980). Quantitative morphological analysis of red blood cells. Blood Cells.

[7] Bacus J.W (1984). Quantitative red cell morphology. Monogr. Clin. Cytol.

[8] Hanion-Lundberg K.M, Kirby R.S, Gandhi S, Broekhuizen F.F (1997). Nucleated red blood cells in cord blood of singleton term neonates. Am. J. Obstet. Gynecol.

[9] Gomez-Villamandos J.C, Bautista M.J, Carrasco L, Caballero M.J, Hervas J, Villeda C.J, Wilkinson P.J, Sierra M.A (1997). African swine fever virus infection of bone marrow:Lesions and pathogenesis. Vet. Pathol.

[10] Karalyan Z, Zakaryan H, Arzumanyan H, Sargsyan K, Voskanyan H, Hakobyan L, Abroyan L, Avetisyan A, Karalova E (2012). Pathology of porcine peripheral white blood cells during infection with African swine fever virus. BMC Vet. Res.

[11] Gómez-Villamandos J.C, Bautista M.J, Sánchez-Cordón P.J, Carrasco L (2013). Pathology of African swine fever:The role of monocyte-macrophage. Virus Res.

[12] Howey E.B, O’Donnell V, de Carvalho Ferreira H.C, Borca M.V, Arzt J (2013). Pathogenesis of highly virulent African swine fever virus in domestic pigs exposed via intraoropharyngeal, intranasopharyngeal, and intramuscular inoculation, and by direct contact with infected pigs. Virus Res.

[13] Hervás J, Gómez-Villamandos J.C, Méndez A, Carrasco L, Sierra M.A (1996). The lesional changes and pathogenesis in the kidney in African swine fever. Vet. Res. Commun.

[14] Okpara R.A (1985). Normoblasts in peripheral blood of Nigerians:Their clinical significance. J. Natl. Med. Assoc.

[15] Tavassoli M (1975). Erythroblastemia. West. J. Med.

[16] Harvey J.W (2001). Atlas of Veterinary Hematology:Blood and Bone Marrow of Domestic Animals.

[17] Evans E.R (1951). Diagnosis of the hemolytic anemias. Calif. Med.

[18] De Klerk G, Rosengarten P.C, Vet R.J, Goudsmit R (1981). Serum erythropoietin (EST) titers in anemia. Blood.

[19] Richmond T.D, Chohan M, Barber D.L (2005). Turning cells red:Signal transduction mediated by erythropoietin. Trends Cell. Biol.

[20] Tada K, Watanabe Y (1962). Serum enzymes in experimental hemolysis:Aldolase, glucose-6-phosphate dehydrogenase and lactic dehydrogenase in serum of rabbits with acute hemolysis by acetylphenylhydrazine. Tohoku J. Exp. Med.

[21] Kato G.J, McGowan V, Machado R.F, Little J.A, Taylor J, Morris C.R, Nichols J.C, Wang X, Poljakovic M, Morris S.M, Gladwin M.T (2006). Lactate dehydrogenase as a biomarker of hemolysis-associated nitric oxide resistance, priapism, leg ulceration, pulmonary hypertension, and death in patients with sickle cell disease. Blood.

[22] Dunkan J.R, Prasse K.W (1979). Veterinary Laboratory Medicine.

[23] Hess C.E, Ayers C.R, Wetzel R.A, Mohler D.N, Sandusky W.R (1971). Dilutional anemia of splenomegaly:An indication for splenectomy. Ann. Surg.

[24] Wang Z, Vogel O, Kuhn G, Gassmann M, Vogel J (2013). Decreased stability of erythroblastic islands in integrin ß3-deficient mice. Physiol. Rep.

[25] Emilia G, Luppi M, Ferrari M.G, Barozzi P, Marasca R, Torelli G (1997). Hepatitis C virus-induced leuko-thrombocytopenia and haemolysis. J. Med. Virol.

[26] Herry I, Cadranel J, Antoine M, Meharzi J, Michelson S, Parrot A, Rozenbaum W, Mayaud C (1996). Cytomegalovirus-induced alveolar hemorrhage in patients with AIDS:A new clinical entity?. Clin. Infect. Dis.

[27] Cammack N, Gould E.A (1985). Conditions for haemolysis by flaviviruses and characterization of the haemolysin. J. Gen. Virol.

[28] Adu F, Esan J, Baba S.S (1990). Seroepidemiological survey for yellow fever antibodies in domestic animals. Rev. Roum. Virol.

[29] Hod E.A, Zimring J.C, Spitalnik S.L (2008). Lessons learned from mouse models of hemolytic transfusion reactions. Curr. Opin. Hematol.

[30] Xu W.S, Qiu X.M, Ou Q.S, Liu C, Lin J.P, Chen H.J, Lin S, Wang W.H, Lin S.R, Chen J (2015). Red blood cell distribution width levels correlate with liver fibrosis and inflammation:A noninvasive serum marker panel to predict the severity of fibrosis and inflammation in patients with hepatitis B. Medicine (Baltimore).

[31] Sornjai W, Khungwanmaythawee K, Svasti S, Fucharoen S, Wintachai P, Yoksan S, Ubol S, Wikan N, Smith D.R (2014). Dengue virus infection of erythroid precursor cells is modulated by both thalassemia trait status and virus adaptation. Virology.

[32] Breese S.S, Hess W.R (1966). Electron microscopy of African swine fever virus hemadsorption. J. Bacteriol.

[33] Karalova E.M, Voskanian G.E, Sarkisian K.H.V, Abroian L.O, Avetisian A.S, Akopian L.A, Semerdzhian Z.B, Zakarian O.S, Arzumanian G.A, Karalian Z.A (2011). Pathology of lymphoid tissue cells infected by African swine fever virus *in vitro*. Vopr. Virusol.

[34] Zakaryan H, Cholakyans V, Simonyan L, Misakyan A, Karalova E, Chavushyan A, Karalyan Z (2015). A study of lymphoid organs and serum proinflammatory cytokines in pigs infected with African swine fever virus genotype II. Arch. Virol.

[35] Jelkmann W (1998). Proinflammatory cytokines lowering erythropoietin production. J. Interferon. Cytokine. Res.

[36] Wang T, Tu M.F, Zhu J, Zheng W, Shao Z.H (2013). The role of cytokines in lymphoma with anemia. Zhongguo Shi Yan Xue Ye Xue Za Zhi.

[37] Gómez-Villamandos J.C, Hervás J, Méndez A, Carrasco L, Martín de las Mulas J, Villeda C.J, Wilkinson P.J, Sierra M.A (1995). Experimental African swine fever:Apoptosis of lymphocytes and virus replication in other cells. J. Gen. Virol.

